# Age most significant predictor of requiring enteral feeding in head-and-neck cancer patients

**DOI:** 10.1186/s13014-015-0408-6

**Published:** 2015-04-18

**Authors:** Sean Sachdev, Tamer Refaat, Ian D Bacchus, Vythialinga Sathiaseelan, Bharat B Mittal

**Affiliations:** Department of Radiation Oncology, Northwestern University Robert H. Lurie Comprehensive Cancer Center, 251 E. Huron Street LC-178, Chicago, IL 60611 USA; Department of Clinical Oncology, Alexandria University, Alexandria, Egypt

**Keywords:** Head-and-neck cancer, Radiotherapy, Enteral feeding, Swallowing dysfunction

## Abstract

**Background:**

A significant number of patients treated for head and neck squamous cell cancer (HNSCC) undergo enteral tube feeding. Data suggest that avoiding enteral feeding can prevent long-term tube dependence and disuse of the swallowing mechanism which has been linked to complications such as prolonged dysphagia and esophageal constriction. We examined detailed dosimetric and clinical parameters to better identify those at risk of requiring enteral feeding.

**Methods:**

One hundred patients with advanced stage HNSCC were retrospectively analyzed after intensity-modulated radiation therapy (IMRT) to a median dose of 70 Gy (range: 60-75 Gy) with concurrent chemotherapy in nearly all cases (97%). Patients with significant weight loss (>10%) in the setting of severely reduced oral intake were referred for placement of a percutaneous endoscopic gastrostomy (PEG) tube. Detailed DVH parameters were collected for several structures. Univariate and multivariate analyses using logistic regression were used to determine clinical and dosimetric factors associated with needing enteral feeding. Dichotomous outcomes were tested using Fisher’s exact test and continuous variables between groups using the Wilcoxon rank-sum test.

**Results:**

Thirty-three percent of patients required placement of an enteral feeding tube. The median time to tube placement was 25 days from start of treatment, after a median dose of 38 Gy. On univariate analysis, age (p = 0.0008), the DFH (Docetaxel/5-FU/Hydroxyurea) chemotherapy regimen (p = .042) and b.i.d treatment (P = 0.040) (used in limited cases on protocol) predicted need for enteral feeding. On multivariate analysis, age remained the single statistically significant factor (p = 0.003) regardless of other clinical features (e.g. BMI) and all radiation planning parameters. For patients 60 or older compared to younger adults, the odds ratio for needing enteral feeding was 4.188 (p = 0.0019).

**Conclusions:**

Older age was found to be the most significant risk factor for needing enteral feeding in patients with locally advanced HNSCC treated with multimodal treatment. Pending further validation, this would support maximizing early nutritional guidance, targeted supplementation, and symptomatic support for older adults (>60) undergoing chemoradiation. Such interventions and others (e.g. swallowing therapy) could possibly delay or minimize the use of enteral feeding, thereby helping avoid tube dependence and tube-associated long-term physiologic consequences.

## Introduction

The use of radiation therapy with concurrent chemotherapy (CRT) has been well established in the treatment of locally advanced head and neck squamous cell carcinoma (HNSCC) [1-4]. Despite clinical benefits in disease control and overall survival, this combination is one of the most toxic oncologic treatments in use [5,6]. Along with mucositis, xerostomia, and acute pain, impairment of the swallowing mechanism can impede the ability to maintain adequate nutritional intake and hydration.

One method to assist patients through treatment is the use of enteral tube feeding. This can be done with use of nasogastric tubes or more commonly, endoscopically-placed percutaneous tubes that bypass the proximal orodigestive tract and provide intake directly into the stomach or distally [7]. While tube placement typically carries low procedural risk, data suggest that enteral feeding can induce long-term tube dependence and disuse of the swallowing mechanism which has been linked to complications such as prolonged dysphagia and esophageal constriction [8].

For these reasons, in our institution and some others, patients are typically started on treatment without routine prior placement of a feeding tube. Instead, there is close monitoring with serial clinical evaluation and assessment of weight, performance status, and laboratory values. Any significant clinical worsening associated with lack of oral intake (and weight loss) is reason for placement of an enteral feeding tube at that time – the so called “reactive” approach.

Here, in a relatively homogenous cohort of patients with advanced stage HNSCC treated with CRT, we conducted a detailed analysis of clinical and dosimetric parameters to better define factors that could predict requirement for enteral feeding. For patients who are deemed high risk, such data could allow an approach of maximizing targeted nutritional guidance, early supplementation, swallowing therapy and more aggressive symptomatic support. If this could help delay or prevent placement of a feeding tube, it could possibly help avoid potential long-term ramifications of enteral feeding.

## Materials and methods

### Patient selection

One hundred patients with locally advanced stage III and IV HNSCC were consecutively treated with sequential intensity-modulated radiation therapy (IMRT) between 2005 and 2010. Patients were chronologically selected in this period if they had a histopathological diagnosis of squamous cell carcinoma of the head-and-neck region, AJCC group stage III or IV, and were treated with sequential IMRT; they were excluded if they had less advanced disease (i.e. stage I or II) or if they were treated with a different modality (e.g. a combination of 3D-CRT/IMRT). They were also excluded if they had a feeding tube placed prior to treatment. The retrospective data collection and analyses were done per the established standards and approval of the Northwestern University institutional review board (IRB).

### Radiation planning and treatment

Patients underwent treatment simulation with use of an Aquaplast face mask (WFR/Aquaplast Corp., Wyckoff, NJ). Each patient was imaged from the vertex to the carina using 3 mm CT slices with IV contrast administration (unless contraindications existed). The simulation images were then imported into the Pinnacle radiation treatment planning system (Phillips Medical Systems, Madison, WI) for subsequent treatment planning.

The radiographically apparent tumor volume (gross tumor volume – GTV) or surgical bed (in adjuvant cases) was contoured along with adjacent at-risk structures, including the spinal cord, brainstem, oropharynx, parotids, larynx, constrictors, postcricoid esophagus, and cervicothoracic esophagus. Twenty-seven organs at risk (OARs) were routinely contoured on all patients undergoing IMRT for head-and-neck squamous cell cancers. Further details on these OARs have been previously reported by our group [9]. Further details can be obtained upon request. Clinical target volumes (CTV) were created to encompass areas of potential microscopic disease. These included areas at risk of nodal spread plus GTV expansions. CTV_1_ included low and high risk nodal volumes and the GTV, expanded by 1-2 cm. CTV_2_ included high risk nodal volumes plus GTV, expanded by 0.75-1 cm. In adjuvant cases, larger margins were utilized for high risk features like extracapsular extension. CTV_3_ was used for definitive (i.e. non-adjuvant) treatment; it equaled the GTV expanded by 0.5-1 cm. Finally, all CTV volumes underwent a volumetric expansion of 3-5 mm to create planning treatment volumes (i.e. PTV_1_, PTV_2_, and PTV_3_).

IMRT plans were constructed with an inverse planning algorithm designed to concentrate dose and maximize conformity to tumor while reducing exposure to nearby critical structures. Treatment plans underwent iterative optimization to meet certain objectives including that: (1) 95% of the target volume gets the prescribed dose and (2) no hot-spot exceed 110% of the prescription dose.

Treatment was delivered via conventional fractionation using doses of 1.8-2.0 Gy per day except for limited cases on protocol treated with 1.5 Gy twice daily. The median prescription dose was 70 Gy (range: 60-75 Gy) with concurrent chemotherapy delivered in nearly all cases (97%). PTV_1_ was usually treated to 40-50 Gy, PTV_2_ to 55-66 Gy and PTV_3_ to 70-75 Gy.

### Clinical evaluation

Prior to treatment, all patients underwent a comprehensive swallowing study to establish baseline functioning. During treatment, patients underwent scheduled clinical evaluations at least once a week or more frequently if indicated. These evaluations included a physical exam (with performance status evaluation) as well as a review of weekly weight and laboratory values along with trends. Analgesics and other supportive medications were adjusted as necessary. Patients with significant weight loss (>10% of baseline) in the setting of severely reduced oral intake were referred for placement of a percutaneous endoscopic gastrostomy (PEG) tube. This was coordinated with a hospital-based gastroenterology team to avoid any breaks in a patient’s treatment course.

Beyond treatment completion, patients were first seen for follow-up at 4–6 weeks (or sooner if clinically indicated). After that, routine follow-up included an evaluation typically every three months for the first year, every four months for the second year, every six months until year 5 and then annually afterwards. After an initial post-treatment scan (CT or PET/CT) further imaging studies were obtained as needed, typically once per year.

### Dose and volumetric data and statistical analysis

Multiple dosimetric parameters (including mean dose, maximum dose, minimum dose) were obtained for at-risk structures including the oral cavity, oropharynx (including base of tongue), constrictors, postcricoid esophagus, larynx, cervicothoracic esophagus, etc. using the Pinnacle radiation treatment planning system (Phillips Medical Systems, Madison, WI).

Statistical testing and descriptive statistics calculations were done using Stata 13 (Stata Corp, College Station, Texas). Pearson’s coefficient was calculated to assess correlation between continuous variables. Fisher’s exact test was used to evaluate outcome association among nominal variables. The Wilcoxon rank-sum test was used to compare continuous variables between groups. Multivariate analysis was done with logistic regression using variables selected based on the results of the univariate analysis. Receiver operating characteristics testing was done for optimal cut-off analysis and model predictive capability assessment. All tests were two sided and a p-value of 0.05 was considered significant.

## Results

One hundred patients with HNSCC were treated with intensity-modulated radiation therapy (IMRT) between 2005 and 2010 to a median dose of 70 Gy (range: 60-75 Gy) with concurrent chemotherapy in nearly all cases (97%). The median age of the cohort was 55 years (range: 30–89) and 83% of the patients were male. Seventy-six percent of patients had N2-N3 disease. All patients had locally advanced stage III or stage IV disease; 18 (18%) had stage III disease and 82 (82%) had stage IV disease. The median pre-treatment body mass index (BMI) was 28.13 (range: 18.5 - 46.8). No patients required enteral feeding at time of treatment commencement. Three of the 13 patients with larynx cancer had undergone a laryngectomy. Treated sites include cancers of the oral cavity, oropharynx, nasopharynx, hypopharynx, larynx, and of unknown primary. Patients treated with cisplatin were treated either with weekly (40 mg/m^2^) or every-three-week (100 mg/m^2^) dosing depending on the preference on the treating medical oncologist. Cumulative dosing details for each treatment were not available. Table 1 lists patient and tumor characteristics in detail.

**Table 1:** **Patient, tumor and treatment characteristics with univariate analysis**

**Variable**	**Number (%)**	**P Value**
**Age (years)**		
Median	55	0.0008
Range	30-89	
**Sex**		
Male	83 (83)	>0.999
Female	17 (17)	
**Performance Status (ECOG)**		
Normal	66 (66)	>0.999
Inhibited (> = 1)	34 (34)	
**Body-Mass-Index (BMI), pretreatment**		
Median	28.1	0.152
**Smoking**		
None	37 (37)	0.536
<20 pack years	26 (26)	
20 - 40 pack years	25 (25)	
>40 pack years	12 (12)	
**Tumor Site**		
Oral Cavity	4 (4)	0.094
Oropharynx	58 (58)	
Hypopharynx	3 (3)	
Nasopharynx	9 (9)	
Larynx	13 (13)	
Unknown primary	13 (13)	
**T stage (AJCC 7th edition)**		
T0-T2	75 (75)	0.185
T3-T4	25 (25)	
**N stage (AJCC 7th edition)**		
N0-N1	24 (24)	0.184
N2-N3	76 (76)	
**Group stage (AJCC 7th edition)**		
III	18 (18)	0.165
IV (locoregional)	72 (72)	
**Chemotherapy**		
Cisplatin	63 (63)	0.114
DFH (Docetaxel/5-FU/Hydroxyurea)	23 (23)	0.042
Cetuximab or other	11 (11)	>0.999
None	3 (3)	
**Induction?**		
Yes	17 (17)	>0.999
No	83 (83)	
**BID treatment?**		
Yes	21 (21)	0.040
No	79 (79)	
**Modality**		
Definitive	77 (77)	0.614
Adjuvant	23 (23)	

*Abbreviations*: AJCC = American Joint Committee on Cancer, ECOG = Eastern Cooperative Oncology Group.

**Figure 1:**
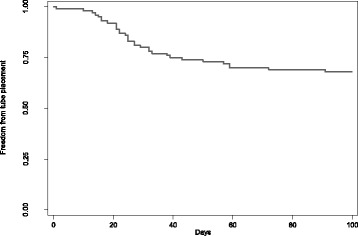
Freedom from tube placement.

Thirty-three percent of patients required placement of an enteral feeding tube. The median time to feeding tube placement was 25 days from start of treatment after a median dose of 38 Gy. The median BMI in the group needing enteral feeding was 29.3 and did not significantly differ from patients who did not need enteral feeding (p = 0.152). Figure 1 display the details of freedom from tube-placement (FFTP) in days. One patient treated with induction chemotherapy with new symptoms of worsening dysphagia underwent tube placement after one fraction; the rest underwent placement after more significant cumulative doses of radiation. After tube placement, 14 (14%) patients had a feeding tube for more than 1 year and of these and only 4 (4%) patients had for more than 2 years.

On univariate analysis, BMI was not correlated with enteral feeding, nor was performance status, smoking status, or gender. Significant variables for tube placement included age (p = 0.0008) and the DFH (Docetaxel – 5-FU – Hydroxyurea) chemotherapy regimen used in limited cases on protocol (p = 0.042). Induction chemotherapy did not predict enteral feeding but b.i.d treatment (when on protocol) was a significant predictor (p = 0.040).

Significant dosimetric parameters as planned included maximum oropharynx dose (p = 0.003), maximum postcricoid esophagus dose (p = 0.043), maximum larynx dose (p = 0.001), mean larynx dose (p = 0.012) maximum constrictor dose (p = 0.002) and mean constrictor dose (p = 0.021). Non-significant parameters included the mean oropharynx dose (p = 0.062), and mean postcricoid esophagus dose (p = 0.10). The cervicothoracic esophagus and parotids were found to have no dosimetric relationship to enteral feeding (in terms of mean dose, max dose, etc.).

**Figure 2:**
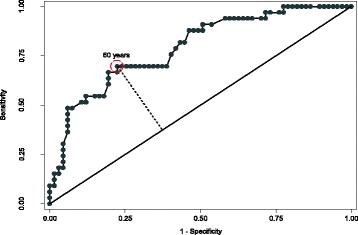
Receiver operating characteristics (ROC) analysis reveals an optimal cut-off of 60 years.

**Figure 3:**
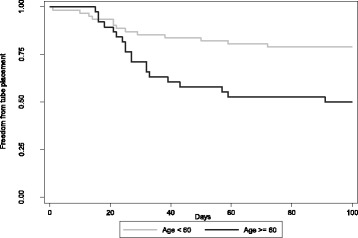
Freedom from tube placement according to age.

On multivariate analysis, after controlling for chemotherapy regimen and b.i.d treatment, age remained the single statistically significant factor in predicting need for enteral feeding (p = 0.003). This did not change when accounting for effects of significant dosimetric (treatment planning) parameters (p = 0.003) with or without including the larynx (p = 0.013) for the three patients who had undergone laryngectomy. Among all patients, age and BMI were not correlated (Pearson’s correlation coefficient; R = 0.0233, p = 0.82) and age remained a highly significant predictor after controlling for BMI (p = 0.003). A receiver operating characteristics (ROC) analysis revealed an optimal age cut-off of 60 as seen in Figure 2. For adults aged 60 or greater compared to younger adults, the odds ratio for needing enteral feeding was 4.188 (95% CI: 1.587-11.16; p = 0.0019). Figure 3 depicts FFTP according to this age cutoff.

## Discussion

The use of CRT in such a physiologically intricate region as the head and neck can lead to difficulties like acute dysphagia and impairment of the swallowing mechanism that can severely limit nutrition and hydration [10,11]. In this setting, adequate intake can be maintained by enteral feeding pursued either via a prophylactic or “reactive” approach. Although the optimal approach has yet to be definitively determined, our institutional approach, similar to that advocated by others [12], favors the “reactive” approach – in which serial clinical assessments help guide need for enteral feeding.

When this can be feasibly pursued (i.e. with enough team resources and a system in place to minimize breaks) the most compelling rationale for eschewing prophylactic tube placement might be avoidance of potential long-term physiologic consequences from disuse of the swallowing mechanism, especially with prolonged tube dependence. Several reports have raised the concern of objectively worse dysphagia and greater need for esophageal dilations in patients who undergo enteral feeding [8,13-15]. In the Radiation Therapy Oncology Group (RTOG) 0129 study, 30% of patients were still tube-dependent at 1 year; in this large cohort, nearly 40% had their feeding tubes placed prophylactically [16].

In this study, we attempted to identify risk factors for enteral feeding in patients without pre-treatment tube placement. If patients at greater risk of enteral feeding could be better identified, they could perhaps be targeted for more early and continued nutritional optimization as well as more aggressive hydration and early symptomatic support (with lower threshold for analgesics and other medications such as oral anesthetic solutions). With pre-treatment swallowing studies, these patients could also be provided early and more aggressive corrective swallowing therapy and exercises [17,18]. While the best way to address the higher risk may need to be determined ahead, these and other potential interventions could possibly delay, minimize the use of, or potentially obviate the need of enteral feeding in more patients. This could also reduce risk from a percutaneous tube placement procedure which, admittedly, is likely safe in experienced hands [19].

Moreover, we examined dosimetric variables (which have also been analyzed and reported by others [20,21]). These planning parameters (e.g. maximum constrictor dose) highlight the importance of minimizing hotspots within critical swallowing structures when feasible (i.e. with optimal tumor coverage). Ultimately, age was found to be the single most significant predictor of enteral feeding, regardless of these dosimetric parameters or other clinical variables including BMI, performance status, smoking status, etc.

Other studies have investigated this question in more heterogeneous cohorts. A study by Mangar and colleagues included 160 patients treated with radiotherapy using a mix of prophylactic and reactive tube placement strategies [22]. In this study, factors associated with enteral feeding included age, performance status, protein/albumin levels, active smoking and body-mass-index. Notably, no patient underwent concurrent chemotherapy and there was no report or analysis of disease stage. There was also no information on radiation technique or dose.

A large 2006 patient survey-based association study also found age to be a significant risk factor for enteral feeding [23]. However, in this study there was no standard approach to feeding tube placement and the cohort included all disease stages (compared to just advanced stage disease in our analysis). Other findings included higher rates of enteral feeding in patients with oropharynx and hypopharynx cancers. No dosimetric parameters were examined and – as a methodological limitation – this survey-based study included patients in any phase of treatment beyond diagnosis.

Al-Othman and colleagues retrospectively reviewed a large number of sequentially treated head-and-neck cancer patients (all stages) treated without IMRT, mostly without chemotherapy from 1983-1997 [24]. In this heterogeneous group, some patients were also treated with Co-60 machines. Important predictors of enteral feeding included age, adjuvant chemotherapy, and presence of neck disease. In contrast, everyone in our cohort had advanced stage disease and almost all patients were treated with chemotherapy, arguably controlling for these factors (while age remained a significant factor).

A common theme from most of these and other studies is that older age remains a significant risk factor for treatment-related oropharyngeal dysfunction, especially for needing enteral feeding. This may hold true even long after treatment. Per an RTOG pooled analysis from trials 9111, 9703 and 9914, risk factors for late pharyngeal toxicity or needing enteral feeding for more than 2 years included older age, advanced T-stage, larynx or hypopharynx primary and neck dissection [6]. Trial 9111 was a study of larynx-preserving radiotherapy while trials 9703 and 9914 investigated chemotherapy options and accelerated radiotherapy, respectively. Notably, in this pooled analysis there was no standard approach for pursuing enteral feeding and only long-term requirement was considered as an endpoint.

In contrast, our data are uniquely derived from a relatively homogenous modern cohort of locally advanced head-and-neck patients treated with concurrent chemotherapy and IMRT, all closely followed with a “reactive” approach to enteral feeding. In a strict sense, for patients treated in this manner, our data would applicably suggest that older age (especially greater than 60) significantly increases risk of enteral feeding. In a broader sense, our study cohort’s composition – patients with advanced stage disease treated with CRT – essentially controls the effects of other significant risk factors; it especially highlights the singular importance of age as an independent risk factor for general treatment-related oropharyngeal dysfunction.

Indeed, studies attempting to correlate swallowing function with age have found numerous physiologic deficits in older subjects. Robbins and colleagues [25] have reported lower lingual pressure generation and pressure reserve among older adults via measurements made during isometric tasks and saliva swallows; others have confirmed these age-related deficits in lingual strength [26]. Aviv et al. have noted deficits in pharyngeal and supraglottic sensitivity with increasing age [27]. Others have found decreased hyoid bone displacement during swallowing as well as problems with pharyngeal strength, transit time, pharyngeal clearance and relaxation of the upper esophageal sphincter [28-30].

A recent prospective study investigated neurophysiologic changes with age, comparing subjects within an age range of 23–37 and 64–83 [31]. In addition to videoflouroscopic monitoring of swallowing biomechanics (with foods of different consistency), investigators examined functional MRI (fMRI) changes during swallowing maneuvers. The older adults had significantly greater delays in pharyngeal response after propulsion of bolus as well as larger amounts of post-swallow residue in the valleculae and upper esophageal sphincter. Importantly, the study’s functional neuroimaging revealed greater recruitment of neurocortical areas in the older subjects, leading to the theory that greater neural involvement was needed to generate greater “effort” for proper swallowing as compared to younger adults.

**Figure 4:**
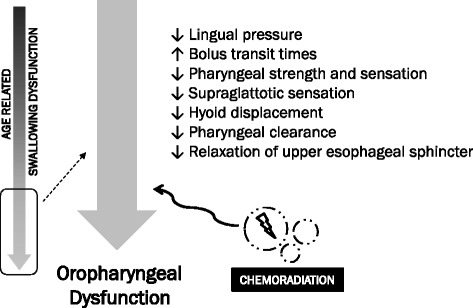
Schematic diagram of age related swallowing dysfunction.

For older patients operating at such a baseline, being exposed to acute treatment-related mucositis and tissue inflammation could mean a critical threshold difference in discomfort and dysphagia, precipitating a need for enteral feeding. Figure 4 highlights this in an illustrative diagram.

While we present a modern cohort of locally advanced head-and-neck patients treated with IMRT-based CRT, as a limitation of our study, the sample size is not large and the treatment delivered is somewhat heterogeneous and thus it is possible that other significant predictors were missed due to limited statistical power. Also, HPV status was not recorded or available on multiple patients and thus was not tested as a possible predictor. Given the significance of age as a parameter, this could be a variable worth examining in future investigations. A few recent studies that have studied this issue in patients with oropharyngeal cancer failed to find a link with age, although the analysis was likely limited by a small number of events in one study (in which patients were treated with chemoradiation) and by a more heterogeneous cohort in the other [32,33]. In the latter study, the authors did notably find a significant reduction in reactive enteral feeding for patients aggressively approached with a proactive swallowing regimen.

In summary, for patients with advanced stage head-and-neck cancer treated with CRT, we found age to be the most significant factor for enteral feeding. Several studies point to age-related physiologic deficits in the swallowing mechanism that might explain this susceptibility. For institutions and clinicians that follow patients in a “reactive” manner for enteral feeding, these data could help physicians selectively target patients for nutritional and symptomatic support and swallowing therapy.

## Abbreviations

HNSCC: Head and neck squamous cell cancer
IMRT: Intensity-modulated radiation therapy
PEG: Percutaneous endoscopic gastrostomy
DFH: Docetaxel/5-FU/Hydroxyurea
BMI: Body-mass index
CRT: Concurrent chemoradiation
IRB: Institutional review board
GTV: Gross tumor volume
CTV: Clinical target volume
PTV: Planning target volume
CT: Computed tomography
PET/CT: Positron emission tomography/computed tomography
FFTP: Freedom from tube-placement
ROC: Receiver operating characteristics
RTOG: Radiation Therapy Oncology Group
fMRI: Functional MRI
